# The Power Gain Difference Method Analysis

**DOI:** 10.3390/s20113018

**Published:** 2020-05-26

**Authors:** Jiří Veselý, Petr Hubáček, Jana Olivová

**Affiliations:** Department of Communication Technologies, Electronic Warfare and Radars, University of Defence in Brno, 66210 Brno, Czech Republic; jiri.vesely@unob.cz (J.V.); petr.hubacek@unob.cz (P.H.)

**Keywords:** received signal strength, localization techniques, Cramer–Rao lower bound, object tracking

## Abstract

In this paper, we propose a new approach to passively locate the 3D position of a signal source. This novel technique, called the power gain difference (PGD), is based only on measuring the received signal strength (RSS) with multiple sensors deployed in the area of interest, while the target transmit power or the equivalent isotropic radiated power (EIRP) is assumed to be unknown. Next, the signal source position is estimated using the knowledge of the ratios of RSS measured on different sensors. First, this article presents the geometric representation and the analytical solution of the model of the PGD technique. Second, the PGD dilution of precision was analyzed in order to gauge the accuracy of measuring the RSS. Finally, a numerical simulation of the performance of the proposed method was carried out and the results are discussed. It seems that the PGD technique has the potential to be a simple and effective solution of the 3D localization problem.

## 1. Introduction

The ability of performing an accurate emitter of a non-cooperative signal location is one of the fundamental functions of many civilian and military reconnaissance systems [1,2,3]. For example, knowing the location of emitters, or targets, is useful for perimeter protection or electronic warfare systems. There are several techniques to estimate the target position based on different information available from measurements performed on received radio frequency (RF) signals. This means that RF based localization systems may use a multitude of different techniques, which include the angle of arrival (AoA) [4,5], received signal strength (RSS), time of arrival (ToA), time difference of arrival (TDoA) [6,7,8], Doppler difference (DD) [9,10], or hybrid location methods [11]. While some localization techniques usually come at a low cost but a lower accuracy, others require complex synchronization schemes, which usually make them more expensive [12,13,14,15,16]. 

The most popular localization methods are based on estimating the RSS. These techniques have the benefit of low implementation cost and the ability to locate targets in both indoor and outdoor environments, etc. On the other hand, the performance of RSS techniques is limited by the quality of the measurement, or estimation, of target-effective radiated power (ERP). The targets’ transmit power and path loss exponent are two parameters that have a significant effect on the performance of the RSS localization techniques. The performance analyses of some RSS-based localization methods [17,18,19] have assumed that the transmit power or path loss exponent is perfectly known. However, this assumption is not suitable for a practical application where the targets do not cooperate with a localization system. Generally, there are two main approaches for using RSS methods for the localization of such targets. The first one consists of using a channel model to establish relations between the measured RSS and the distance sensor–target [20]. The channel model-based methods are not very accurate, particularly due to the fact that multipath propagation, fading, and shadowing affect the estimated received signal strength. The RSS can also be employed in localization techniques based on a “power map” [21,22], which represents the second approach. These techniques consist of creating a “power map” with the observed RSS from different receiving sensors at different positions. This map is later used during the localization step to find the closest point (in the RSS space) to the unknown one.

In this paper, we focused on the first approach. In particular, we proposed only using the ratios of the measured RSS, rather than their absolute values, on four receiving sensors to calculate the 3D position of a signal source, for example, a down-link transmitter of unmanned aerial vehicle (UAV). This approach is not conditioned upon knowing the signal source ERP, or an estimation of the path loss exponent [23], which is the most significant benefit of the proposed localization technique in comparison with the traditional positioning ones for RSS-based localization [24,25]. The proposed technique also provides a closed-form solution of the target localization problem. The method was tested through some numerical simulations.

## 2. The Geometric Representation and the Analytical Solution of the Power Gain Difference (PGD) Technique Model

First, we describe a typical arrangement for the proposed localization method in this section. This is followed by the mathematical model of the localization technique including an analytical solution.

**Figure 1:**
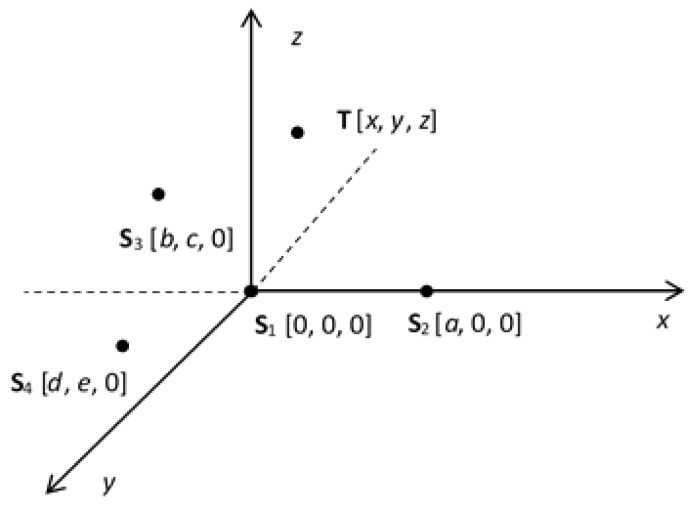
The sensor network and the target arrangement.

Let us consider a network composed of a set of fixed receiving sensors. In this example, there are four sensors, **S**_1_ … **S**_4_, and one target **T**. The arrangement of such a network is shown in Figure 1.

The measured received power (in watts) by the sensor *i* is given by:

(1) $$P_{i}=\frac{1}{\left\{{\left(x-x_{i}\right)}^{2}+{\left(y-y_{i}\right)}^{2}+{\left(z-z_{i}\right)}^{2}\right\}}\cdot Q$$

where $x_{i}$, $y_{i}$, $z_{i}$ are the sensor coordinates and Q includes the target-transmitted power, the gain of sensor antennas, the gain of target antenna, the wavelength, and all losses of the target–sensor communication channel. The measured powers on particular sensors are written according to Equation (1) as:

(2) $$P_{1}=\frac{1}{\left\{x^{2}+y^{2}+z^{2}\right\}}\cdot Q$$

(3) $$P_{2}=\frac{1}{\left\{{\left(x-a\right)}^{2}+y^{2}+z^{2}\right\}}\cdot Q$$

(4) $$P_{3}=\frac{1}{\left\{{\left(x-b\right)}^{2}+{\left(y-c\right)}^{2}+z^{2}\right\}}\cdot Q$$

(5) $$P_{4}=\frac{1}{\left\{{\left(x-d\right)}^{2}+{\left(y-e\right)}^{2}+z^{2}\right\}}\cdot Q$$

Then, the power ratios, related to the power received by the first sensor, are:

(6) $$J_{1}=\frac{P_{2}}{P_{1}}=\frac{x^{2}+y^{2}+z^{2}}{x^{2}-2\cdot a\cdot x+a^{2}+y^{2}+z^{2}}=\frac{K}{K-2\cdot a\cdot x+a^{2}}$$

(7) $$J_{2}=\frac{P_{3}}{P_{1}}=\frac{x^{2}+y^{2}+z^{2}}{x^{2}-2\cdot b\cdot x+b^{2}+y^{2}-2\cdot c\cdot y+c^{2}+z^{2}}=\frac{K}{K-2\cdot b\cdot x+b^{2}-2\cdot c\cdot y+c^{2}}$$

(8) $$J_{3}=\frac{P_{4}}{P_{1}}=\frac{x^{2}+y^{2}+z^{2}}{x^{2}-2\cdot d\cdot x+d^{2}+y^{2}-2\cdot e\cdot y+e^{2}+z^{2}}=\frac{K}{K-2\cdot d\cdot x+d^{2}-2\cdot e\cdot y+e^{2}}$$

where

(9) $$K=x^{2}+y^{2}+z^{2}$$

By using Equation (6) and applying some algebra, the equation for the *x* target coordinate is obtained:

(10) x=A+B·K

where $A=\frac{a}{2}$ and $B=\frac{J_{1}-1}{2\cdot a\cdot J_{1}}$.

A similar approach can then be to applied to Equations (7) and (8) with the goal of solving the *y* target coordinate. Then,

(11) y=C+D·K

(12) y=E+F·K

where $C=\frac{-2\cdot b\cdot A\cdot J_{2}+b^{2}\cdot J_{2}+c^{2}\cdot J_{2}}{2\cdot c\cdot J_{2}}$, $D=\frac{J_{2}-1-2\cdot b\cdot B\cdot J_{2}}{2\cdot c\cdot J_{2}}$, $E=\frac{-2\cdot d\cdot A\cdot J_{3}+d^{2}\cdot J_{3}+e^{2}\cdot J_{3}}{2\cdot e\cdot J_{3}}$, and $F=\frac{J_{3}-1-2\cdot d\cdot B\cdot J_{3}}{2\cdot e\cdot J_{3}}$.

By comparing Equations (11) and (12), the solution of *K* is given by

(13) $$K=\frac{E-C}{D-F}$$

Finally, the *z* target coordinate, using Equation (9), is expressed by

(14) $$z=\pm \sqrt{K-x^{2}-y^{2}}$$

Equations (10), (11) or (12) and (14) represent the target 3D position. It is clear that the *z* coordinate can take on two values. This means that the PGD technique is ambiguous in such an sensor arrangement. From a practical point of view, the derived algorithm of the proposed method was applied in this way. First, the received powers *P*_1_ to *P*_4_ were measured and the ratios *J*_1_ to *J*_3_ were determined. Next, all variables *A*, *B*, *C*, *D*, *E*, *F*, and *K* were computed. Finally, the target coordinates *x*, *y*, *z* were found.

## 3. The PGD Technique Accuracy Analysis

### 3.1. The Theoretical Basis of the Accuracy Analysis

In many localization applications, the Cramér–Rao Lower Bound (CRLB) is used to assess their localization accuracy. It is well known that CRLB sets a lower limit for the variance of any unbiased estimate of any unknown parameter [26]. The same approach was chosen for analyzing the accuracy of the proposed method. Generally, the *CRLB* is calculated from the inverse of the Fisher Information Matrix (FIM) **I**. Thus:

(15) $$var\left(\hat{x}\right)\geq CRLB\left(x\right)=I^{-1}\left(x\right)$$

where x is an unknown parameter and $\hat{x}$ is its unbiased estimate. 

In the proposed PGD method, the target location vector T=[x,y,z] is the unknown parameter, or the parameter of interest, and $\hat{T}$ is an estimate of it. The vector $\hat{T}$ is obtained by using the vector of measured powers $\hat{P}=\left[\hat{P_{1}},\hat{P_{2}},\hat{P_{3}},\hat{P_{4}},\;\right]$ and knowledge of the sensor arrangement (expressed by vectors of sensor coordinates **S**_1_ to **S**_4_). Generally, it can be written as:

(16) $$\hat{T}=f\left(\hat{P},S_{1..4}\right)$$

It should be noted that the estimates of particular target coordinates are specifically described in Equations (10), (11), (12), and (14) and that all elements of vector $\hat{P}$ are Gaussian random variables with dispersion $\sigma_{Pi}^{2}$.

In accordance with [27,28] and by using Equation (16), it is possible to express the CRLB by:

(17) $$CRLB\left(T\right)=\left[\frac{\partial f\left(\hat{P},S_{1..4}\right)}{\partial P}\right]\cdot C_{p}\left(\hat{P}\right)\cdot {\left[\frac{\partial f\left(\hat{P},S_{1..4}\right)}{\partial P}\right]}^{T}$$

where $C_{p}\left(\hat{P}\right)$ is the covariance matrix of the vector $\hat{P}$. If the received power measurements on particular sensors are independent as well as in the PGD method, the matrix $C_{p}\left(\hat{P}\right)$ becomes a diagonal matrix in the following form:

(18) $$C_{p}\left(\hat{P}\right)=\left[\begin{array}{cccc} \sigma_{P1}^{2} & 0 & 0 & 0\\ 0 & \sigma_{P2}^{2} & 0 & 0\\ 0 & 0 & \sigma_{P3}^{2} & 0\\ 0 & 0 & 0 & \sigma_{P4}^{2} \end{array}\right]$$

Next, it is appropriate to introduce the Jacobian matrix **J**.

(19) $$J\left(\hat{P}\right)=\left[\frac{\partial f\left(\hat{P},S_{1..4}\right)}{\partial P}\right]=\left[\begin{array}{ccc} \frac{\partial x\left(\hat{P},\;S_{1..4}\right)}{\partial P_{1}} & \ldots & \frac{\partial x\left(\hat{P},\;S_{1..4}\right)}{\partial P_{4}}\\ \frac{\partial y\left(\hat{P},\;S_{1..4}\right)}{\partial P_{1}} & \ldots & \frac{\partial y\left(\hat{P},\;S_{1..4}\right)}{\partial P_{4}}\\ \frac{\partial z\left(\hat{P},\;S_{1..4}\right)}{\partial P_{1}} & \ldots & \frac{\partial z\left(\hat{P},\;S_{1..4}\right)}{\partial P_{4}} \end{array}\right]$$

The $J\left(\hat{P}\right)$ consists of real values of partial derivatives of the function $f\left(P,S_{1..4}\right)$ with respect to variables *P*_1_ to *P*_4_ for the given measured value of the vector $\hat{P}$. In fact, the function $f\left(P,S_{1..4}\right)$ is expressed by Equations (10), (11) or (12) and (14). Therefore, the partial derivatives of ones are used in the practical calculation of the Jacobian matrix. An example of finding a partial derivative is given in Appendix A.

Finally, the covariance matrix of the proposed localization technique is:

(20) $$CRLB\left(T\right)=C\left(T\right)=J\left(\hat{P}\right)\cdot C_{p}\left(\hat{P}\right)\cdot J{\left(\hat{P}\right)}^{T}$$

The proof of Equation (20) is given in Appendix B.

Thus, the defined covariance matrix is positive-semidefinite and represents a confidence region that includes the “true” value of the target position with a certain probability level [29]. In 3D localization applications, the covariance matrix describes the error ellipsoid with a probability of target occurrence of 0.213. According to [30], the center of the error ellipsoid is at the estimate of the target position. The lengths of the axes of the ellipsoid *A_e_*, *B_e_*, *C_e_* are proportional to the eigenvalues *λ*_1_, *λ*_2_, *λ*_3_ of the covariance matrix and the directions of these axes are given by the eigenvectors of one. 

### 3.2. The Results of the Error Analysis of the PGD Method

Before presenting some results of an error analysis of the PGD method, we would like to present a calculation of the covariance matrix for a simple example of using the PGD method. Let us suppose a network composed of four receiving sensors with coordinates **S**_1_[0, 0, 0], **S**_2_[200 m, 0, 0], **S**_3_[140 m, −140 m, 0] and **S**_4_[−140 m, −140 m, 0]. The antenna gain of each sensor is *G_s_* = 7 dB and its receiver is able to measure power with a standard deviation equal to 0.1 dB. The target position is **T** [20 m, 70 m, 70 m] and it transmits a signal with a power of *P_t_* = 0.5 W and a wavelength of 0.1 m. The gain of the target antenna is *G_t_* = 0 dB. 

**Figure 2:**
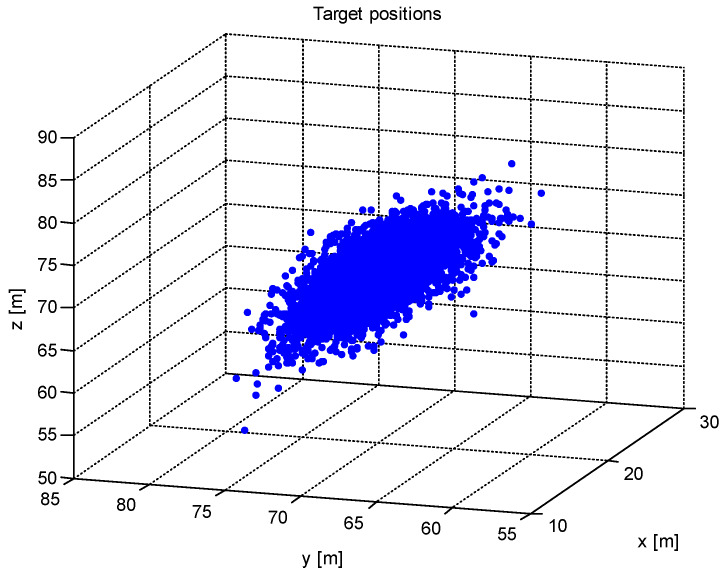
The measured target positions.

Then, after making *N* = 1000 measurements, the mean values of the received powers on the particular sensor are *P*_1_ = −48.1 dBm, *P*_2_ = −54.2 dBm, *P*_3_ = −53.5 dBm and *P*_4_ = −56.7 dBm. This corresponds to the estimated position of the target $\hat{T}$[20.1 m, 70.1 m, 69.5 m]. The covariance matrix is

(21) $$CRLB\left(T\right)=\left[\begin{array}{ccc} 3.8271 & 2.4464 & -3.2820\\ 2.4464 & 8.7276 & -9.2234\\ -3.2820 & -9.2234 & 15.8069 \end{array}\right]$$

and the lengths of the axes of the error ellipsoid are *A_e_* = 0.77 m, *B_e_* = 0.86 m, *C_e_* = 2.40 m. The measured target positions are shown in Figure 2.

The lengths of the axes of the error ellipsoid indicate that the PGD localization method is able to provide an estimation of the target position with an error to the order of meters. It is clear that the above-mentioned example does not evaluate the overall performance of the proposed technique from a localization accuracy perspective. 

**Figure 3:**
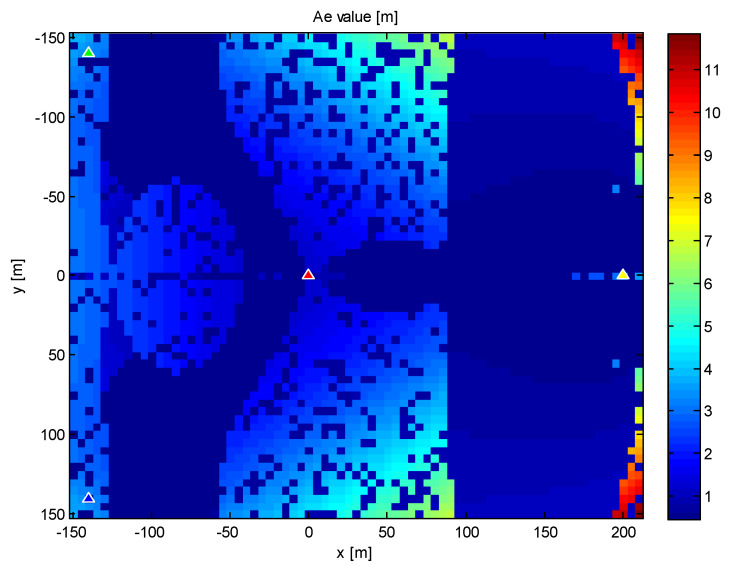
The values of the *A_e_* parameter.

Therefore, we will try the same network of sensors and the same target as in the previous example. Next, we can define an area by the coordinates x∈〈−150 m, 210 m〉, y=〈−150 m,150 m〉, and *z* = 70 m. Finally, the *CRLB* is computed for all possible target locations within this area with a step of 5 m, thereby providing an “accuracy map” of the PGD method. The values of the axis length *A_e_* of the appropriate error ellipsoids are shown in Figure 3, providing one possible way to interpret the accuracy map.

From a practical point of view, it is often sufficient to determine the maximum and minimum values of the error ellipsoid axes. In our example, they were:

- *A_emin_* = 0.47 m, *A_emax_* = 5.84 m,
- *B_emin_* = 0.46 m, *B_emax_* = 4.09 m,
- *C_emin_* = 0.47 m, *C_emax_* = 8.97 m.

This way of calculating the covariance matrix facilitates the detailed description of the accuracy of the PGD method for both an arbitrary sensor network arrangement and an arbitrary target position in an area of interest. Equally, the knowledge of the covariance matrix can be used to optimize the sensor network arrangement by the criterion of the maximal allowed error. The accuracy can also be evaluated according to the quality of the received power measurement.

## 4. The Simulation of the Method Performance

**Figure 4:**
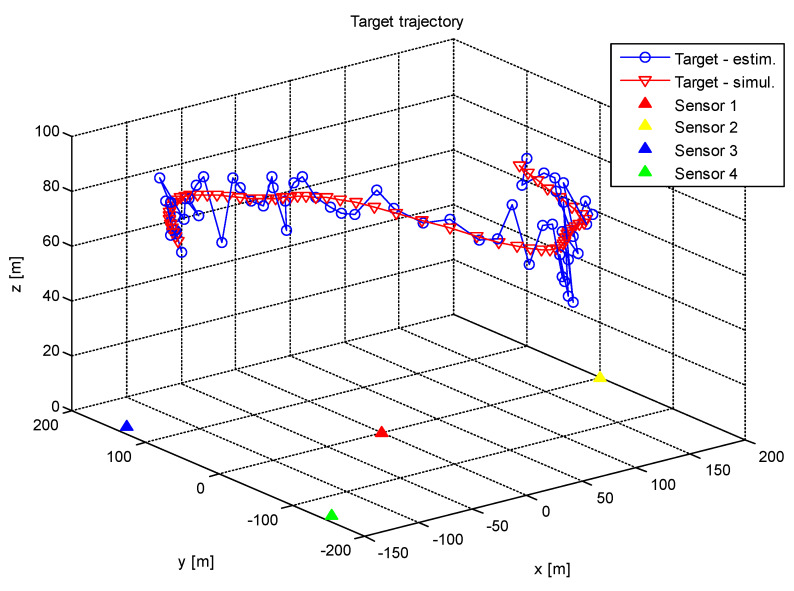
The target trajectories.

In order to validate our approach to the accuracy analysis of the PGD method, we carried out a simulation that was almost the realistic operation of one. We utilized the same sensor infrastructure that was described in Section 3. The target was moving and its trajectory was randomly placed into the area of interest (it was identical to the area defined in Section 3). The *z* coordinate of the target trajectory was set to the value 70 m. This situation is shown in Figure 4.

Approximately 60 measurements of received powers on the particular sensors were made during the target motion. The standard deviation of power measurement was set to 0.1 dB. Thus, there were 60 estimates of the target position on the output of the simulation algorithm. These estimates were compared with the corresponding actual target locations. This means that the overall range deviation of the target was calculated using

(22) $$\Delta R=\|\hat{T}-T\|$$

**Figure 5:**
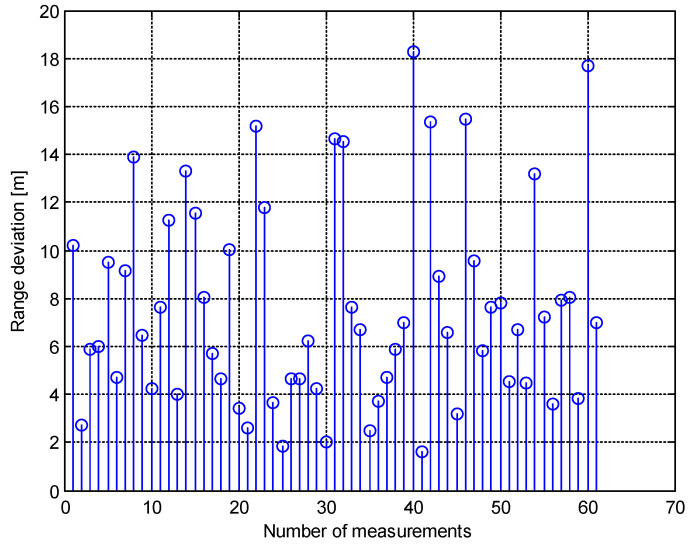
The range deviation of the target (simulation 1).

The graph of the ΔR is shown in Figure 5. 

The simulation also provided some numerical results. The most important were the mean value of the range deviation, the standard deviation of one, and the maximum and minimum values of ΔR. These were

- $\Delta R_{mean}=7.6\;m$,
- $\Delta R_{std}=4.2\;m$,
- $\Delta R_{max}=18.2\;m$,
- $\Delta R_{min}=1.6\;m$.

Next, the same simulation was performed, but the *z* coordinate of the target trajectory was set to the new value of 100 m and the standard deviation of the power measurement was set to 0.2 dB. Then, the results of this simulation were as follows:

- $\Delta R_{mean}=16.3\;m$,
- $\Delta R_{std}=9.5\;m$,
- $\Delta R_{max}=44.7\;m$,
- $\Delta R_{min}=2.9\;m$.

**Figure 6:**
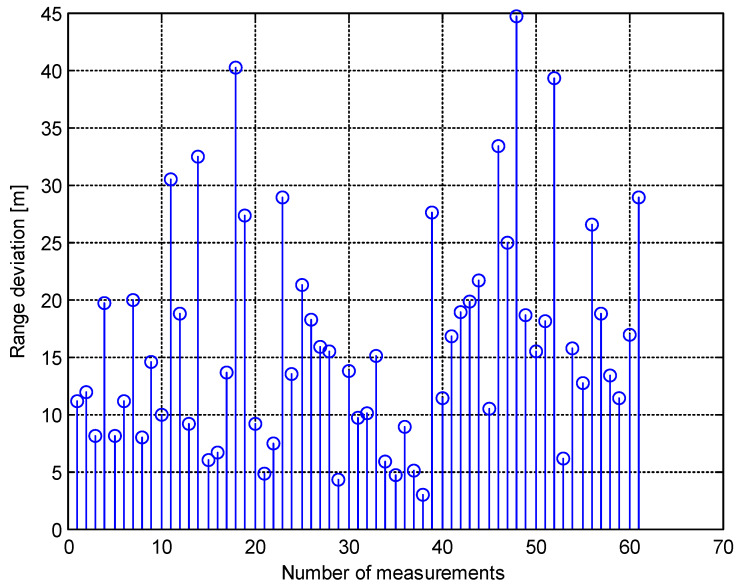
The range deviation of the target (simulation 2).

The graph of the ΔR is shown in Figure 6.

The achieved simulation results indicate that the proposed PGD method is able to provide an estimate of the target position with an acceptable error. It seems that the determination of the target *z* coordinate was at least accurate, as seen in Figure 4. It is also clear that the overall range deviation ΔR increased with the increase in the standard deviation of the power measurement and the target altitude.

## 5. Discussion

**Figure 7:**
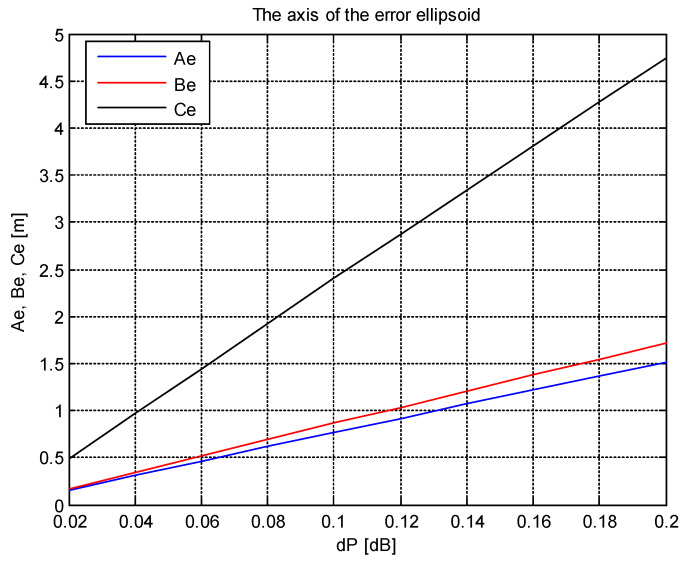
The lengths of axes of the error ellipsoid depending on the standard deviation of the power measurement.

In terms of the practical use of the proposed method, the accuracy of measuring the received power is a critical point of this method. In view of the example given in Section 3, it turns out that this accuracy must be at the level of 1.10^−9^ W (this corresponds to measuring the received power with a standard deviation equal to 0.1 dB). It is evident that such accuracy can only be achieved by measuring the received power over a certain integration time [31]. Reducing the requirement for the accuracy of the received power measurement (i.e., an increase in the standard deviation of the power measurement) leads to a linear increase in the axis lengths of the error ellipsoid. This situation is shown in Figure 7 for the same sensor network arrangement and target position as in the previous example.

One of the most interesting effects of the presented method is its possibility of estimating the target EIRP (using the calculated target position and Equations (2), (3), (4), or (5)). The knowledge of the target EIRP can be used for the recognition of the adaptive power management of the target transmitter or for cooperation with the standard 3D RSS method. Suppose that the target EIRP is estimated by the PGD method. Then, the cooperation with the standard RSS method can be as follows:

- The target position can be estimated by the standard RSS method in the case that only three receiver sensors are irradiated by the target (for example, due to the shadowing effect).
- The target position can be estimated by the fusion of the localization data that the PGD and the RSS methods provide in the case that four or more sensors are irradiated.

Only the issue of cooperation between the PGD and standard RSS methods will be the content of our future work.

## 6. Conclusions

The presented power gain difference method can be used for transmitter target location estimation. The main advantage of the proposed method is the independency on a priori knowledge of transmitted power or EIRP, making this method usable for a non-cooperating target. In this paper, the model and computational algorithms were derived and the target coordination accuracy were analyzed for selected scenarios. The main limitation of this method is that the accuracy of the measured RSS (in this example 0.1 dB) has to be measured accurately. This measuring accuracy cannot be based on absolute error, but on relative error (i.e., compared to all receivers). This is a very strict requirement not from the measuring equipment perspective, but as the transmitter and receiving antenna difference from an ideal omnidirectional antenna. These differences are critical mainly in low altitudes, or rather low elevation angles, due to the ground effect of the antenna radiating diagram of the receiving antennas. A similar effect will be in the transmitter and the effect of the fuselage of the target on the transmitting antenna radiating pattern. Nevertheless, these effects are similar in the standard RSS location method, so these methods (RSS and PGD) can be used combined to increase the accuracy, robustness, and integrity in order to locate targets with an unknown EIRP or the adaptive calibration of the RSS method itself.

## Appendix A

The Jacobian matrix **J** is defined as:

(A1) $$J\left(\hat{P}\right)=\left[\frac{\partial f\left(\hat{P},S_{1..4}\right)}{\partial P}\right]=\left[\begin{array}{ccc} \frac{\partial x\left(\hat{P},\;S_{1..4}\right)}{\partial P_{1}} & \ldots & \frac{\partial x\left(\hat{P},\;S_{1..4}\right)}{\partial P_{4}}\\ \frac{\partial y\left(\hat{P},\;S_{1..4}\right)}{\partial P_{1}} & \ldots & \frac{\partial y\left(\hat{P},\;S_{1..4}\right)}{\partial P_{4}}\\ \frac{\partial z\left(\hat{P},\;S_{1..4}\right)}{\partial P_{1}} & \ldots & \frac{\partial z\left(\hat{P},\;S_{1..4}\right)}{\partial P_{4}} \end{array}\right]$$

The example of a calculation of partial derivatives is as follows.

For example, the *x* target coordinate is obtained by the equation

(A2) x=A+B·K

where $A=\frac{a}{2}$, $B=\frac{J_{1}-1}{2\cdot a\cdot J_{1}}$, and $K=\frac{E-C}{D-F}$ are respective substitutes.

Then, the partial derivate of *x* with respect to *P*_1_ is

(A3) $$\frac{\partial x\left(\hat{P},\;S_{1..4}\right)}{\partial P_{1}}=\frac{\partial A}{\partial P_{1}}+\frac{\partial B}{\partial P_{1}}\cdot K+B\cdot \frac{\partial K}{\partial P_{1}},$$

where the partial derivatives of substitutes *A*, *B*, and *K* with respect to *P*_1_ are

(A4) $$\frac{\partial A}{\partial P_{1}}=0,$$

(A5) $$\frac{\partial B}{\partial P_{1}}=\frac{\frac{\partial J_{1}}{\partial P_{1}}\left(2\cdot a\cdot J_{1}\right)-2\cdot a\cdot J_{1}\cdot \frac{\partial J_{1}}{\partial P_{1}}\left(J_{1}-1\right)}{{\left(2\cdot a\cdot J_{1}\right)}^{2}},$$

(A6) $$\frac{\partial K}{\partial P_{1}}=\frac{\left(\frac{\partial E}{\partial P_{1}}-\frac{\partial C}{\partial P_{1}}\right)\cdot \left(D-F\right)-\left(E-C\right)\cdot \left(\frac{\partial D}{\partial P_{1}}-\frac{\partial F}{\partial P_{1}}\right)}{{\left(D-F\right)}^{2}}.$$

In view of the fact that variables (or substitutes) *J*_1_, *C*, *D*, *E* and *F* are also a function of *P*_1_, it is necessary to calculate their partial derivatives. For example, if

(A7) $$J_{1}=\frac{P_{2}}{P_{1}},$$

then the corresponding partial derivate is

(A8) $$\frac{\partial J_{1}}{\partial P_{1}}=-\frac{P_{2}}{P_{1}^{2}}.$$

The remaining partial derivatives of the Jacobian matrix can be derived in the same way.

## Appendix B

An estimate $\hat{T}=\left[\hat{x},\hat{y,}\hat{z}\right]$ of the vector of the true target position T=[x,y,z] can be written as:

(A9) $$\hat{T}=f\left(\hat{P},S_{1..4}\right)=T+\delta_{T}$$

where $\delta_{T}=\left[\delta_{x},\delta_{y},\delta_{z}\right]$ is the vector of errors in the determination of the particular target coordinates. The function $f\left(\hat{P},S_{1..4}\right)$ is specifically described by the particular functions:

(A10) $$\hat{x}=f_{1}\left(\hat{P},S_{1..4}\right)$$

(A11) $$\hat{y}=f_{2}\left(\hat{P},S_{1..4}\right)$$

(A12) $$\hat{z}=f_{3}\left(\hat{P},S_{1..4}\right)$$

which are equivalent to Equations (10), (11), and (12). The Taylor series expansions of $f_{1}$, $f_{2}$, and $f_{3}$ around point $\hat{P}$, after deleting all terms higher than the linear ones, are expressed as:

(A13) $$f_{1}\left(P,S_{1..4}\right)=f_{1}\left(\hat{P},S_{1..4}\right)+\frac{\partial f_{1}\left(\hat{P},S_{1..4}\right)}{\partial P}\cdot \delta_{P}$$

(A14) $$f_{2}\left(P,S_{1..4}\right)=f_{2}\left(\hat{P},S_{1..4}\right)+\frac{\partial f_{2}\left(\hat{P},S_{1..4}\right)}{\partial P}\cdot \delta_{P}$$

(A15) $$f_{3}\left(P,S_{1..4}\right)=f_{3}\left(\hat{P},S_{1..4}\right)+\frac{\partial f_{3}\left(\hat{P},S_{1..4}\right)}{\partial P}\cdot \delta_{P}$$

where $\delta_{P}=\left[\delta_{P1},\delta_{P2},\delta_{P3},\delta_{P4}\right]$ is the vector of errors in the received power measurement.

If $x=f_{1}\left(P,S_{1..4}\right)$, $y=f_{2}\left(P,S_{1..4}\right)$ and $z=f_{3}\left(P,S_{1..4}\right)$ are the true particular target coordinates, then Equations (A13)–(A15) can be written as

(A16) $$\delta_{x}=\frac{\partial f_{1}\left(\hat{P},S_{1..4}\right)}{\partial P_{1}}\cdot \delta_{P1}+\frac{\partial f_{1}\left(\hat{P},S_{1..4}\right)}{\partial P_{2}}\cdot \delta_{P2}+\frac{\partial f_{1}\left(\hat{P},S_{1..4}\right)}{\partial P_{3}}\cdot \delta_{P3}+\frac{\partial f_{1}\left(\hat{P},S_{1..4}\right)}{\partial P_{4}}\cdot \delta_{P4}$$

(A17) $$\delta_{y}=\frac{\partial f_{2}\left(\hat{P},S_{1..4}\right)}{\partial P_{1}}\cdot \delta_{P1}+\frac{\partial f_{2}\left(\hat{P},S_{1..4}\right)}{\partial P_{2}}\cdot \delta_{P2}+\frac{\partial f_{2}\left(\hat{P},S_{1..4}\right)}{\partial P_{3}}\cdot \delta_{P3}+\frac{\partial f_{2}\left(\hat{P},S_{1..4}\right)}{\partial P_{4}}\cdot \delta_{P4}$$

(A18) $$\delta_{z}=\frac{\partial f_{3}\left(\hat{P},S_{1..4}\right)}{\partial P_{1}}\cdot \delta_{P1}+\frac{\partial f_{3}\left(\hat{P},S_{1..4}\right)}{\partial P_{2}}\cdot \delta_{P2}+\frac{\partial f_{3}\left(\hat{P},S_{1..4}\right)}{\partial P_{3}}\cdot \delta_{P3}+\frac{\partial f_{3}\left(\hat{P},S_{1..4}\right)}{\partial P_{4}}\cdot \delta_{P4}$$

To put this development into matrix form for easier manipulation, define

(A19) $$\left[\begin{array}{ccc} \frac{\partial f_{1}\left(\hat{P},\;S_{1..4}\right)}{\partial P_{1}} & \ldots & \frac{\partial f_{1}\left(\hat{P},\;S_{1..4}\right)}{\partial P_{4}}\\ \frac{\partial f_{2}\left(\hat{P},\;S_{1..4}\right)}{\partial P_{1}} & \ldots & \frac{\partial f_{2}\left(\hat{P},\;S_{1..4}\right)}{\partial P_{4}}\\ \frac{\partial f_{3}\left(\hat{P},\;S_{1..4}\right)}{\partial P_{1}} & \ldots & \frac{\partial f_{3}\left(\hat{P},\;S_{1..4}\right)}{\partial P_{4}} \end{array}\right]\cdot \left[\begin{array}{c} \delta_{P1}\\ \vdots \\ \delta_{P4} \end{array}\right]=\left[\begin{array}{c} \delta_{x}\\ \delta_{y}\\ \delta_{z} \end{array}\right]$$

Then Equation (A19) can be written as

(A20) $$J\left(\hat{P}\right)\cdot \delta_{P}=\delta_{T}$$

where $J\left(\hat{P}\right)$ is the Jacobian matrix.

The goal is to find the error covariance matrix C(T) with entries $c_{i,j}=E\left\{\delta_{i}\delta_{j}\right\}$, where E{} denotes the statistical expectation. Using Equations (A19) and (A20), the covariance matrix can be written as

(A21) $$C\left(T\right)=E\left\{\left[\begin{array}{c} \delta_{x}\\ \delta_{y}\\ \delta_{z} \end{array}\right]\cdot {\left[\begin{array}{c} \delta_{x}\\ \delta_{y}\\ \delta_{z} \end{array}\right]}^{T}\right\}=E\left\{J\left(\hat{P}\right)\cdot \left[\begin{array}{c} \delta_{P1}\\ \vdots \\ \delta_{P4} \end{array}\right]\cdot {\left[\begin{array}{c} \delta_{P1}\\ \vdots \\ \delta_{P4} \end{array}\right]}^{T}\cdot J{\left(\hat{P}\right)}^{T}\right\}=J\left(\hat{P}\right)\cdot E\left\{\left[\begin{array}{c} \delta_{P1}\\ \vdots \\ \delta_{P4} \end{array}\right]\cdot {\left[\begin{array}{c} \delta_{P1}\\ \vdots \\ \delta_{P4} \end{array}\right]}^{T}\right\}\cdot J{\left(\hat{P}\right)}^{T}$$

The expression $E\left\{\left[\begin{array}{c} \delta_{P1}\\ \vdots \\ \delta_{P4} \end{array}\right]\cdot {\left[\begin{array}{c} \delta_{P1}\\ \vdots \\ \delta_{P4} \end{array}\right]}^{T}\right\}$ represents the covariance matrix $C_{p}\left(\hat{P}\right)$ of the vector $\hat{P}$.

If the received power measurements on particular sensors are independent and identically distributed as well as in the PGD method, the matrix $C_{p}\left(\hat{P}\right)$ becomes the diagonal matrix in the following form:

(A22) $$C_{p}\left(\hat{P}\right)=\left[\begin{array}{cccc} \sigma_{P1}^{2} & 0 & 0 & 0\\ 0 & \sigma_{P2}^{2} & 0 & 0\\ 0 & 0 & \sigma_{P3}^{2} & 0\\ 0 & 0 & 0 & \sigma_{P4}^{2} \end{array}\right]$$

where $\sigma_{Pi}^{2}$ is the dispersion of particular elements of the vector $\hat{P}$.

Finally, the covariance matrix of the proposed localization technique is

(A23) $$CRLB\left(T\right)=C\left(T\right)=J\left(\hat{P}\right)\cdot C_{p}\left(\hat{P}\right)\cdot J{\left(\hat{P}\right)}^{T}$$
